# Evaluation of Serum & Salivary IgA in Patients with Type 1 Diabetes

**DOI:** 10.1371/journal.pone.0122757

**Published:** 2015-04-13

**Authors:** Akefeh Ahmadiafshar, Mahmood Reza Mohsenifard, Saeideh Mazloomzadeh

**Affiliations:** 1 Metabolic Diseases Research Center, Zanjan University of Medical Sciences, Zanjan, Iran; 2 Social Determinants of Health Research Center, Zanjan University of Medical Sciences, Zanjan, Iran; 3 Mousavi Hospital, Zanjan University of Medical Sciences, Zanjan, Iran; University of Michigan Medical School, UNITED STATES

## Abstract

**Background:**

Diabetes mellitus is a common immune mediated disorder. The aim of the present study is to evaluate the level of serum and salivary IgA levels in patients with Type 1 diabetes.

**Material and Method:**

In this case control study, serum and salivary IgA levels of patients with diabetes type 1 and similar non diabetes subjects were measured. Age, gender, duration of diabetes and the last HbA1c level of diabetic patients were also studied. Data was analyzed by SPSS software.

**Results:**

Two hundred and fifty subjects (126 diabetics and 124 non diabetics) were enrolled in the study. The mean value of serum IgA in patients with Type 1 Diabetes and controls was 1.77± 1.55 g/lit and 2.39± 1.52 g/lit, respectively. The mean salivary IgA level in diabetics and controls was 276 ± 162.5 40 μg/ml and 129 ± 112.2 40 μg/ml, respectively. Selective IgA deficiency was detected in two (1.6%) and three(2.4%)cases of diabetic and control group; respectively (p=0.68). We found low salivary IgA level in 44.4% diabetic and 33.9% control (p=0.08). There was no significant correlation between serum and salivary IgA level. There was also significant association between serum IgA levels with age. Salivary IgA was significantly correlated with HbA1c level. But considering gender, duration of diabetes we didn’t find any association.

**Conclusion:**

We didn't find any significant difference in serum and salivary IgA level among diabetic and non diabetics and also, no association between serum and salivary IgA levels.

## Introduction

Selective IgA deficiency is the most common primary immunodeficiency. Its incidence varies depending on ethnic background from 1/143 to 1/185000 in different geographic areas [1]. It is usually asymptomatic. However, these patients are more susceptible to frequent infections, autoimmune disorders, gastrointestinal diseases and atopy. It seems that, the increased frequency of infections associated with IgA deficiency could therefore precipitate autoimmune disease such as systemic lupus erythematus, Graves' disease, celiac, recurrent parotiditis, inflammatory bowel syndrome, Crohn's disease, juvenile idiopathic arthritis and type 1 diabetes [1–5]. Some studies showed the role of some related genes that was found in IgA deficiency and some autoimmune disorders.[6]. Furthermore, there was little evidence about the role of secretory IgA as a first line defense in prevention of these disorders. One study showed lower Secretory IgA in diabetic patients, [7] but some studies did not show any significant changes in salivary IgA of diabetes patients.[8,9]. On the other hand, association between serum and secretory IgA remain to be explored. The aim of this study was to investigate and compare serum and salivary IgA level in type I diabetic patients and non diabetic controls.

## Materials and Methods

### Subjects

This prospective case control study was conducted in Diabetes Clinic of Valie Asr hospital and registry of Diabetic Society of Zanjan, Zanjan, Iran, between April 2012 to February 2013. Patients with type1 diabetes were selected and consecutively enrolled to study. The diagnosis of diabetes was made on the basis of diagnostic criteria of The American Diabetes Association [10] Patients with any diabetic complications and subjects with ages lower than 5 years, having Acute or chronic infectious, inflammatory, endocrine and immunologic disorders and those who had received anticonvulsive drugs, penicilamine and non steroidal anti inflammatory drugs at least 4 weeks before study were excluded. The control group was selected from healthy accompanying non relative voluntaries or medical staff of the hospital.

### Specimen collection, handling and testing

This Study was approved by Ethics Research Committee of Zanjan University of Medical Sciences and at the beginning of study all the participants or their parents assigned the informed consent form. After that, blood and salivary samples were collected from patients and controls. In each case 2 ml of whole blood was collected by venipuncture, centrifuged and serum samples were stored at -70°C for serum IgA determination. Serum IgA concentration were determined by nephelometry method (MININEPH TM Human IgA Kit, the binding Site Ltd, Birmingham, UK). The saliva samples were also obtained by spitting out to a glass tube and centrifuged at 3000 rpm for 15 minutes to remove any particulate material and then supernatants were immediately frozen at -20°C and stored for secretory IgA assessment. Quantitative assessment of IgA in saliva was determined by ELISA method (Dia Metra, Milano, Italy). Information on age, gender, duration of disease and the last HbA1c was compiled using questionnaires. Selective IgA deficiency was considered based on serum IgA level lower than 0.07 gr/l [11]. Normal range of salivary IgA was 40–170 on the basis of Diametra kit (Dia Metra, Milano, Italy), thus, the cut off point for IgA deficiency in serum and saliva was ≤0.07 gr/l and ≤40 μg/ml, respectively.

### Statistical analysis

The Kolmogorov-Smirnov test was used to evaluate the distribution of quantitative variables. Values were expressed as number (percentage), and mean±standard deviation, as appropriate. Comparisons were performed by chi-square test for categorical variables, independent *T*-test for normally distributed, and Mann–Whitney test for non-normally distributed. Pearson's and Spearman correlation coefficient were calculated for assessment of correlation between quantitative and qualitative variables, respectively. A multivariate logistic regression model was used to examine the association between the independent and dependent variables. Differences with a p value less than 0.05 were accepted as statistically significant. All statistical analyses were performed using the SPSS PC version 16.0 computer software program for Windows (SPSS, Chicago, IL, USA).

## Results

One hundred and twenty six type 1 diabetic patients and 124 healthy controls were recruited to study. The mean age of patients and controls was 24.2 ±13.1 and 29.8 ± 11.7 respectively (p = 0.0001). The male to female ratio of patients and controls was similar (p = 0.62). The mean serum IgA concentration in Diabetic patients was significantly lower than control group (p<0.0001), but after adjusting for age using logistic regression this association was not significant.

We didn't find any significant difference in salivary IgA levels in patients and controls (p = 0.724). (Table 1)

**Table 1:** Characteristics, Serum and Salivary IgA levels and frequency of Low serum and salivary IgA in Type 1 Diabetic patients and non diabetic subjects.

Variety	Diabetics	No diabetics	P value
Male/female	52/74	64/60	0.26
Age, Year (mean± SD)	24.2± 13.1	29.8± 11.7	0.0001
†HbA1c	7.64± 1.14	-	-
Duration of Diabetes, Year	7.7± 6.4	-	-
Serum IgA, g/l	1.77± 1.55	2.39± 1.52	<0.0001*
Salivary IgA μg/ml	276 ± 162.5	129 ± 112.2	0.724
Selective IgA Deficiency Number (%)	2 (1.6%)	3(2.4%)	0.68
Low salivary IgA Number (%)	56 (44.4)	42 (33.9)	0.08

^†^This didn't measured in non diabetic group

* This association was not significant after adjusting for age using logistic regression.

Two (1.6%) and three(2.4%)cases in diabetic and control group; respectively had selective IgA deficiency (p = 0.68). Furthermore, the frequency of low salivary IgA in patients (44.4%) and control (33.9%) was not statistically significant (p = 0.08) (Table 1).

The correlation of serum IgA with age in control subjects was significant (r = 0.301, = 0.001) but its relation with gender was not significant (r = -0.1, P = 0.29).

We found significant correlation between serum IgA and age in diabetics (r = 0.24, p = 0.007) but its association with gender, duration of disease and HbA1c in diabetic group was not statistically significant. (Table 2)

**Table 2:** Correlation between serum IgA and other variables in diabetic individuals.

Variables	r	P
** Age**	0.239	0.007*
** ** † **Gender**	0.13	0.16
** Duration of Diabetes**	0.07	0.44
** HbA1c**	0.04	0.732
** Salivary IgA**	0.1	0.25

^†^Correlation coefficient was calculated by Spearman

*P Value<0.05

The correlation between Salivary IgA with HbA1c in diabetics was significant (p = 0.003) but its association with age, gender, duration of disease was not statistically significant. (Table 3)

**Table 3:** Correlation coefficient between salivary IgA and other Variables in diabetic individuals.

Variables	r	P
** Age**	0.16	0.07
** ** † **Gender**	0.003	0.96
** Duration of Diabetes**	0.07	0.45
** HbA1c**	0.34	0.003*
** Serum IgA**	0.1	0.25

^†^Correlation coefficient was calculated by Spearman.

*P Value<0.05.

One subject with IgA deficiency in diabetics and all 3 IgA deficient cases in control group, had low salivary IgA level, however, the correlation between serum and salivary IgA in both groups was not significant (r = 0.1, P = 0.25).

## Discussion

In this study the prevalence of IgA deficiency in our study population (diabetics and non-diabetics) was about 2%, which was at least two fold higher than other studies.[12–17]

Although, some studies showed higher rate of IgA deficiency in diabetic patients [2,18], in our study the frequency of selective IgA deficiency in patient and control groups was similar. Genetic susceptibility and ethnic background or the method of selection or sampling of study population might be explained these differences. Therefore further investigations in this area could be clarified it.

Although, several studies showed an elevated mean of serum IgA concentration in type 1 and type2 diabetic patients specially for those with complications compared to controls.[18–22] in our study the mean of serum IgA concentration in diabetics was lower than control. Absence of infection, complications or other autoimmune disorders in our patients could be explained this contrary result.

Serum IgA is in monomeric form, whereas two third of IgA located in mucosal areas is secretary IgA and has dimeric form. It has an important role in protection of mucociliary areas and plays as a barrier against pathogenic organisms, antigens and even allergens, thus any defect in this defense system could be an important risk factor for development of autoimmune disorders and allergy or increasing the susceptibility to infection. [1,23] Some evidence support that there is a complex interaction between the intestinal microorganisms and the immune system in the maintenance of the normal immune homeostasis in the gut. Changes in gut micro flora in susceptible individuals could lead to alternation in the gut immune system such as increased gut permeability, impaired tolerance to food, intestinal inflammation and Islet cell destruction [24–26]. Our study did not show any significant difference in salivary IgA in diabetic and control group, furthermore there was no significant correlation between serum and salivary IgA levels in study groups. Rashkova and colleagues showed similar results [9], however several studies demonstrated higher salivary IgA in diabetic patients. [8,21,27] Bhuyan and coworker showed lower level of salivary IgA in diabetes specially uncontrolled diabetic patients, in spite of the higher serum IgA levels in diabetic individuals. [18].

The regulation of secretion and synthesis of secretory IgA not only is dependent on antigenic stimulation, but is also under strong neuroendocrine control. Thus, alterations in neuroendocrine function such as; stress, exercise, pregnancy, menstrual cycle, may affect salivary IgA levels [23,28]. Secretory IgA level also directly increased with age even in healthy subjects. [29] In our study the serum IgA level was significantly associated with age of individuals. (Table 2), However salivary IgA had no significant correlation with age. (Table 3)

In our study about one third and one forth of diabetic and non diabetic subjects, respectively had low salivary IgA (salivary IgA <40 μg/ml) level. According to different method of collection, induction of saliva and doubt about the standard level of IgA in saliva, there was not documented data about low salivary IgA in literature. [30] Lamb and coworkers found a significant association between cow's milk protein intake and islet cell autoimmunity and type1 diabetes in children with low/moderate genetic type 1 diabetes risk [31]. Therefore it seems that, mucosal immunity might play an important protective role against diabetes.

Immunologic status of diabetic patients specially antibody production may be impaired in high glucose concentration. [32] We found significant correlation between salivary IgA and HbA1c levels. However, serum IgA levels did not have significant correlation with HbA1c and the period of disease. Our study finding was compatible with study of Sayarifard and colleagues, which did not found any association between serum IgA and HbA1c levels. [17]

In conclusion, this study showed the lower mean serum IgA levels in diabetic patients but after adjustment with age it was not significant. In addition, the salivary IgA levels in patients and controls did not differ significantly. There was no significant correlation between serum and salivary IgA. We also found higher frequency of selective IgA deficiency and low salivary IgA in our study subjects. Therefore, further investigations for finding causal factors and assessment of health status of our population might be meaningful. With respect to the risk of potential anaphylactic reaction to transfusion of blood product, recognition and screening of these patients could be beneficial.
